# Predictive Factors of Resilience in Early Childhood Care Professionals

**DOI:** 10.3390/healthcare13010081

**Published:** 2025-01-04

**Authors:** Sofía Gómez-Herrera, Maria Auxiliadora Robles-Bello, David Sánchez-Teruel, Aziz Sarhani-Robles

**Affiliations:** 1 Department of Psychology, University of Jaen, 23071 Jaen, Spain; sgh00013@red.ujaen.es; 2 Faculty of Psychology, University of Granada, 18012 Granada, Spain; 3 Faculty of Medicine, University of Granada, 18012 Granada, Spain; asarhanir@correo.ugr.es

**Keywords:** resilience, early intervention, healthcare professionals, personal distress, use of emotion, emotional intelligence, empathy

## Abstract

**Background/Objectives:** Early childhood intervention professionals have higher rates of work-related stress and burnout compared to other health professionals. Furthermore, this is exacerbated by exposure to negative emotions, the stigma associated with mental health, and even the stress experienced by families due to the impact of having a child with a developmental disability. The aim of this study was to determine whether emotional intelligence and empathy were able to predict resilience in early childhood care professionals. **Methods:** The total sample consisted of 139 people (128 women and 11 men, with a mean age of 32.69 and SD 9.72) who were divided into two groups: high resilience (M = 35.85; SD = 3.64) and low resilience (M = 20.74; SD = 3.84). **Results:** The results showed significant differences between the two groups in self and others’ emotional appraisal, use and regulation of emotion, perspective taking, and personal distress, with a positive relationship between resilience and all sub-dimensions of emotional intelligence and perspective taking and a negative relationship with personal distress. In addition, a predictive model of resilience in early childhood professionals was found with empathic concern, personal distress, and use of emotion. **Conclusions:** This study is useful to start investigating psychological aspects related to early intervention and its professionals in order to consolidate a resilient workforce.

## 1. Introduction

Mental health has been defined by the World Health Organization (WHO) [1] as a state of wellbeing in which a person is aware of his or her abilities, copes adequately with problems, and is productive and able to contribute to his or her community. However, the Pan-American Health Organisation (PAHO) [2] reports in its World Report on Mental Health that one in eight people in the world suffers from a mental disorder, at great cost to the public health of countries. Nevertheless, on average, less than 2% of the general government budget is allocated to mental health, indicating that there are few resources to address this situation. For this reason, a Comprehensive Mental Health Action Plan (2013–2030) [3] has been implemented, which highlights the need to increase the number of health professionals specialising in mental health worldwide. However, the mental health of healthcare workers is also a cause for concern. The COVID-19 pandemic highlighted the need to address the emotional wellbeing and stress issues associated with this type of workplace [4]. This problem is not unique to the recent pandemic but has been present in society throughout time. Several researchers [5,6] have concluded that medical professionals have higher rates of depression than the general population from the beginning of their careers and even have a higher risk of suicide. These factors are further compounded for mental health professionals by additional challenges such as societal misconceptions about mental health, frequent exposure to intense emotions, working with people in crisis, managing suicide risk, and maintaining extensive documentation [7]. Protecting the health and safety of healthcare workers is a win–win situation. The World Health Assembly (2021) endorsed the need to develop the World Action Plan for Patient Safety (2021–2030) [8]. One of the strategic goals of this plan is to inspire, educate, prepare, and protect all healthcare workers to contribute to the design and delivery of safe systems of care. This includes the development of specific training programmes for staff working in high-risk areas, such as intensive care and emergency services. This is because the health workers involved in these services can suffer lasting psychological damage and high levels of guilt and self-criticism [9].

This is the case of early childhood intervention (ECI) professionals. ECI professionals in Spain, including paediatricians, psychologists, educators, physiotherapists, speech therapists, and social workers, focus on the treatment of children ages 0–6 years with developmental disorders or at risk of developing them [10,11]. This role is further complicated by the family stress caused by the impact of raising a child with these conditions, which can indirectly affect the emotional wellbeing of professionals [12]. Despite these challenges, there are currently no national strategies that specifically address the emotional wellbeing of ECI professionals. Research by Scanlan and Still [13] shows that job satisfaction among health and social care professionals, such as doctors, nurses, occupational therapists, psychologists, and social workers, is strongly associated with turnover intentions and burnout. However, studies focusing specifically on ECI professionals remain scarce, particularly those exploring protective factors that promote resilience in these demanding roles [14].

Resilience has been proposed as an element capable of reducing burnout in addition to empowering professionals to gain self-confidence and self-regulation [15]. Many authors affirm that a competent, motivated, and resilient workforce is necessary to achieve industry goals [16]. Mealer also concluded that health professionals with higher levels of resilience have a greater ability to cope with work stress and prevent burnout. Some researchers have linked resilience to emotional intelligence (EI), suggesting that emotionally intelligent people are more resilient, better able to adapt to changes in stressful conditions, and see stress as a challenge rather than a threat [17,18]. Therefore, emotional intelligence could be considered a protective factor in resilience and even as a predictor of employment success [19,20,21]. Nightingale [22] concluded that if healthcare professionals with higher emotional intelligence were more compassionate, empathetic, resilient, affectionate, and able to manage the emotions of others, they would be better able to care for themselves and their patients. There is also evidence that empathy, self-compassion, and/or resilience may prevent burnout in health professionals [23]. However, few studies have analysed the relationship between these variables and the quality of life of professionals in stressful situations.

Therefore, the general aim of the present study is to know whether emotional intelligence and empathy are able to predict resilience in early childhood care professionals. In addition, a specific aim is to test the relationship between these factors and resilience. Finally, it will be determined which of these factors are most important in terms of predictive ability. It is expected that participants who scored high on resilience will also score high on emotional intelligence and empathy, and vice versa, and that emotional intelligence will have a greater predictive capacity for resilience.

## 2. Materials and Methods

### 2.1. Participants

The sample consisted of 207 people, 21 men and 186 women, between 23 and 63 years old (M = 33.33; SD = 9.89). Inclusion criteria for this study were (1) voluntary participation, (2) signing the informed consent, (3) completing all tests administered, and (4) working or having worked in the field of early childhood care. On the other hand, the exclusion criteria were (1) being a family member of an early childhood attention user and (2) being under 18 years of age. After applying these criteria, the resulting sample was divided into 139 people, of which 128 were women (92.30%) and 11 were men (7.70%), with an age ranging from 23 to 59 years old (M = 32.69; SD = 9.72). The final sample was then divided into two groups. The criterion used was ± 3 SDs above the sample mean, as the questionnaire used (CD-RISC-10) has no cut-off points [24,25]. The result was two subgroups: high resilience with 72 people ages 23 to 59 years (M = 35.85; SD = 3.64) and low resilience with 67 people ages 23 to 49 years (M = 20.74; SD = 3.84). The sociodemographic characteristics of the final sample and each of the subsamples are shown in Table 1.

### 2.2. Evaluation Measures

The ad-hoc questionnaire measured the variables of gender, age, professional experience in early childhood intervention, and profession.

Connor and Davidson’s CD-RISC-10 questionnaire measured resilience. This questionnaire consists of two basic versions, one with 25 items and a second with 10 items. For this research, we used the Spanish adaptation of the 10-item brief questionnaire [26], which assesses resilience in a global way. This adaptation had a Cronbach’s alpha of 0.85, the same validity as in the original scale. The Cronbach’s alpha for the current sample was 0.92.

The Wong Law Emotional Intelligence Scale (WLEIS-S) Questionnaire assesses emotional intelligence [27]. The Spanish version was used. It is a self-report measure based on Salovey and Mayer’s theory of emotional intelligence. It consists of 16 items, four factors, and a total index of emotional intelligence. The factors are “self emotional appraisal”, “others emotional appraisal”, “use of emotion” and “regulation of emotion”. The validity of the Spanish adaptation of this questionnaire is high, reaching 0.91 at the global level. In addition, the internal consistency of each dimension was 0.79, 0.81, 0.81, and 0.84, respectively [28,29]. As for the validity of the sample in question, it was found to be 0.94 overall, with Cronbach’s alphas of 0.92, 0.87, 0.84, and 0.86 for each dimension, respectively.

The Spanish adaptation of the Interpersonal Reactivity Index (IRI 2004) by Davis [30] measures empathy [31]. This test consists of 28 items and is grouped into four subscales: “perspective taking”, “empathic concern”, “personal distress”, and “fantasy” in empathy. The reliability of the Spanish version of this questionnaire was 0.56, 0.65, 0.64, and 0.70, respectively. In the present study, Cronbach’s alphas were obtained for “perspective taking” (0.70), “empathic concern” (0.44), “personal distress” (0.77), and “fantasy” (0.71).

### 2.3. Design and Procedure

This research is based on a descriptive and predictive design using a selective and cross-sectional methodology. First, the necessary documentation to guarantee the confidentiality of the data was sent to the ethics committee of the university of one of the authors, which issued a favourable report. An online questionnaire was then developed using Google Forms. In it, informed consent was attached as a mandatory prerequisite for the performance of psychometric tests; without accepting and reading the consent, the questionnaire could not be accessed to complete the tests. The link was then shared on social networks. The link was active for two months. After the deadline, the test was inactivated, and the data were collected. The results were then coded for analysis. This design and procedure were carried out throughout 2023.

### 2.4. Data Analysis

The programme used was SPSS version 23.0 (Statistical Package for Social Sciences). First, a descriptive analysis was carried out. Second, Student’s t-statistic was used to compare the resulting arithmetic means. Thirdly, the relationship between each of the independent variables and the dependent variable was examined. Finally, Forward Stepwise Multiple Linear Regression was used to test which subdimensions of emotional intelligence and empathy were more predictive of resilience in the overall sample. The required level of significance for all tests was *p* ≤ 0.05, *p* ≤ 0.01, or *p* ≤ 0.001

## 3. Results

### 3.1. Statistical Descriptions

Looking at the differences between the LR and HR groups, statistically significant differences were observed for all variables examined except for fantasy and empathic concern. See Table 2.

### 3.2. Correlational Analysis

The results show that there was a significant linear correlation between resilience and all sub-dimensions of emotional intelligence. At the same time, only the negative linear correlation between resilience and personal distress was significant. See Table 3.

### 3.3. Multiple Linear Regression Model

First, a multiple regression fit analysis was carried out, in which the data did not seem to show multicollinearity, except for the emotional intelligence scale, whose VIF associated with the total variable and the evaluation of others’ emotions exceeded 10 points. Additionally, the VIF associated with the rest of the EI variables was around moderate values (variance inflation factor (VIF) = 5.35 for the use of emotion and 7.35 for the regulation of emotion) [32]. The results of the multiple regression analysis showed that some of the psychosocial variables predicted the level of resilience in the total sample of participants (Table 4). In fact, model 2, which was carried out using forward stepwise multiple regression, was the most significant, explaining 61% of the variance (*F*_(2, 36)_ = 30.89, MCR = 28.06; R^2Adj^ = 0.61). In terms of model parameters, the variables fantasy, perspective taking, empathic concern, self-emotion appraisal, other’s emotional appraisal, regulation of emotion, and total emotional intelligence are not included, as none of them significantly predicted resilience. The only significant parameters associated with the point of origin (14.62) were the slope of the regression line for the variables use of emotion (β = 0.63; IC (95%) = 0.68–1.47) and personal distress (β = −0.28; IC (95%) = −0.80–0.07).

## 4. Discussion

The overall aim of this research was to find out whether emotional intelligence and empathy were predictive of resilience, hypothesising that participants with higher levels of resilience would also have higher scores on emotional intelligence and empathy.

The results showed that all participants scored high on these three variables, with the exception of emotion use and regulation, which were the lowest scoring variables in the overall sample. In line with Salovey and Mayer’s definition [29], this would be associated with a less resilient pattern, as people with low regulation of emotions recover more slowly from personal distress and, according to Newman, a person follows a resilient pattern when he or she activates positive adaptive responses to adversity [33]. The results of this study could be explained by the fact that early childhood care professionals are specialists in the mental health of others, and this involves the development of transversal competences, such as the ability to perceive, understand, and express one’s own and other’s emotions. However, a deficit is observed in the use and regulation of these emotions, which could be explained by the lack of coping strategies in such a demanding environment as early childhood care.

One of the specific aims was to examine the relationship between the variables of resilience, emotional intelligence, and empathy. In this case, it was concluded that there were positive correlations between resilience and emotional intelligence, agreeing with Schneider, who argued that emotionally intelligent people have a more resilient capacity, facilitating a better adaptation to changes in stressful conditions and viewing stress as a challenge rather than a threat [18]. Resilience and empathy were also found to be positively correlated with perspective taking and negatively correlated with personal distress. Given that perspective taking can be seen as vicariously experiencing what is happening to others and seeking to understand it, it could be seen as predictive of resilience, understood as a self-regulatory mechanism that protects personal systems from negative outcomes during difficult life stages and learns from them [34]. The relationship between personal distress and resilience was also found to be negative [35]. However, there were negative correlations between use and regulation of emotion and personal distress, demonstrating Salovey and Mayer’s as well as Davis’ definitions, as it is understood that a person with optimal use and regulation of emotion is able to channel their emotions into constructive activities and recover more quickly from personal distress [29,30]. There were also positive correlations between all subscales of the WLEIS and perspective taking, as these concepts are related, as predicted by Alecsiuk [36]. This conclusion is in line with Mayer’s concept of emotional intelligence, which classifies EI into perceiving, using, understanding, and managing emotions, with empathy being part of the ability to perceive the emotions of others and being able to manage one’s own emotions; therefore, a person would develop social actions congruent with the emotional state of others, thus helping to adapt to the emotional state of others [37,38]. As observed in the results, an early intervention professional is able to understand and express their own and others’ emotions, learns from experiences, and isolates themselves from negative experiences in order to increase their resilience. However, this research found linear correlations between the cognitive components of empathy but no such correlation with the emotional part of empathy.

Finally, the predictive ability of the different variables and the corresponding subscales of emotional intelligence and empathy in relation to the level of resilience was examined, with the hypothesis that emotional intelligence would be the most predictive of resilience. Finally, the analyses showed that empathic concern and personal distress were the variables that were most predictive of resilience, meaning that a non-resilient person would tend to feel excessive feelings of compassion, concern, and caring for the distress of others as well as personal distress when observing the negative experiences of others. The World Health Organization (WHO) has already stated that healthcare workers can suffer lasting psychological impairment and high levels of guilt in their work [9]. The use of emotions was also added to these variables, with the assumption that a resilient person would be able to channel their emotions into constructive activities and personal performance, aside from the discomfort of observing the negative experiences of others. This conclusion is supported by Connor and Davidson’s definition of resilience as a personality trait that enables individuals to thrive in the face of adversity and as a self-regulatory mechanism that protects personal systems from negative outcomes during difficult life stages [24,33].

These findings are important in order to be able to offer courses that enhance emotional intelligence and perspective taking in order to increase the resilience of early childhood professionals. This has a double benefit, as promoting the emotional wellbeing of professionals will result in higher quality personalised care for users. This study also opens up a new line of research to create a scientific basis exclusively related to this type of professional.

However, it is important to note that this study had several limitations. Firstly, the use of self-report measures to assess the variables may have introduced social desirability bias. Future research could address this limitation by using other types of measures. Secondly, it would be worthwhile to develop this study further using a longitudinal design, incorporating a treatment focused on emotional intelligence and empathy. Additionally, the inclusion of coping strategies could prove beneficial. Finally, other researchers are invited to replicate this study by comparing early childhood professionals with non-health professionals.

## 5. Conclusions

This research has concluded that people who are able to channel their emotions into constructive activities other than excessive concern for others and the personal discomfort this causes are more resilient than those who do not meet these characteristics. The protective factors of resilience can therefore be enhanced to create a motivated and resilient workforce, improving the quality of patient care and the emotional wellbeing of the workforce itself.

In conclusion, early childhood care is an underdeveloped branch of public healthcare. We see this research as the beginning of a long study of the characteristics of early childhood care and the mental health of its professionals [14].

## Figures and Tables

**Table 1:** Sociodemographic data.

Variable	Total	LR n (%)	HR n (%)	x^2^	*p*
Sex					
Woman	128 (92.30)	60 (89.50)	68 (95)	0.42	0.52
Man	11 (7.70)	7 (10.50)	4 (5)		
Age					
20–30 years	75 (53.80)	39 (57.90)	36 (50)	3.62	0.31
31–40 years	36 (25.60)	22 (31.60)	14 (20)		
41–50 years	18 (12.80)	7 (10.50)	11 (15)		
Over 51 years old	11 (7.70)	0	11 (15)		
Profession					
Psychology	57 (41)	21 (31.60)	36 (50)	1.40	0.50
Physiotherapy	39 (28.20)	21 (31.60)	18 (25)		
Logopedia	43 (30.80)	25 (36.80)	18 (25)		
Work Experience					
1 year or less	39 (28.20)	21 (31.60)	18 (25)	0.66	0.88
Between 1 and 5 years (inclusive)	50 (35.90)	21 (31.60)	29 (40)		
Between 6 and 10 years (inclusive)	32 (23.10)	18 (26.30)	14 (20)		
More than 11 years	18 (12.80)	7 (10.50)	11 (15)		
Total	139 (100)	67 (48.70)	72 (51.30)		

LR = low resilience group; HR = high resilience group; n = sample; % = sample percentage; x^2^ = chi-square test of independence; *p* = significance.

**Table 2:** Descriptive data on variables studied.

Variables	Total		LR		HR		*t*
	M	SD	M	SD	M	SD	
EI							
SEA	21.13	5.82	17.95	6.44	24.15	2.96	−3.83 ***
OEA	21.38	4.55	18.58	4.74	24.05	2.19	−4.59 ***
UOE	19.51	4.95	15.84	3.04	23	3.74	−6.54 ***
ROE	18.69	5.30	15.26	3.36	21.95	4.74	−5.06 ***
Total	80.72	17.64	67.63	13.04	93.15	11.28	−6.55 ***
IRI2004							
Perspective taking	25.31	5.10	23	5.17	27.50	4.05	−3.03 **
Fantasy	21.77	5.26	21.89	6.04	21.65	4.57	0.14 ^ns^
Empathic concern	26.38	3.85	26.47	4.34	26.30	3.44	0.14 ^ns^
Personal distress	16.33	5.44	18.84	5.03	13.95	4.80	3.11 **
CD-RISC-10	28.49	8.50	20.74	3.84	35.85	3.65	−12.61 ***

Note: Example of EI items: I always set goals for myself and then try my best to achieve them. Example of IRI2004 items: I feel apprehensive and uncomfortable in emergency situations. Example of CD-RISC-10 items: I am able to adapt to change. LR = low resilience group; HR = high resilience group; *t* = Student’s *t* statistic; M = arithmetic mean; SD = standard deviation; EI = emotional intelligence; SEA = self-emotional appraisal; OEA = others’ emotional appraisal; UOE = use of emotion; ROE = regulation of emotion; ** *p* < 0.01; *** *p* < 0.001; ^ns^ = not significant.

**Table 3:** Pearson’s linear correlation.

	EI SEA	EI OEA	EI UOE	EI ROE	EI T	IRI TP	IRI F	IRI PE	IRI MP	CD-RISC-10
EI SEA	1	0.85 **	0.49 **	0.54 **	0.85 **	0.50 **	0.10	0.43 **	−0.22	0.49 **
EI OEA	0.85 **	1	0.63 **	0.61 **	0.90 **	0.58 **	0.18	0.31	−0.24	0.61 **
EI UOE	0.49 **	0.63 **	1	0.75 **	0.83 **	0.37 *	−0.02	−0.02	−0.47 **	0.76 **
EI ROE	0.54 **	0.61 **	0.75 **	1	0.85 **	0.43 **	−0.08	0.03	−0.33 *	0.64 **
EI T	0.85 **	0.90 **	0.83 **	0.85 **	1	0.55 **	0.05	0.23	−0.36 *	0.72 **
IRI TP	0.50 **	0.58 **	0.37 *	0.43 **	0.55 **	1	0.18	0.29	−0.13	0.47 **
IRI F	0.10	0.18	−0.02	−0.08	0.05	0.18	1	0.32 *	0.33 *	−0.04
IRI PE	0.43 **	0.31	−0.02	0.03	0.23	0.29	0.32	1	0.14	−0.12
IRI MP	−0.22	−0.24	−0.47 **	−0.33 *	−0.36 *	−0.13	0.33 *	0.14	1	−0.57 **
CD-RISC-10	0.49 **	0.61 **	0.76 **	0.64 **	0.72 **	0.47 **	−0.04	−0.12	−0.57 **	1

EI SEA = self-emotional appraisal; EI OEA = others’ emotional appraisal; EI UOE = use of emotion; EI ROE = regulation of emotion; EI T = total emotional intelligence; IRI TP = perspective taking; IRI F = fantasy; IRI PE = empathic concern; IRI MP = personal distress; * *p* < 0.05; ** *p* < 0.01

**Table 4:** Predictive models of emotional intelligence and empathy in early child care professionals in relation to levels of resilience.

Models and Variables	VIF	R	R^2^	R^2Adj^	ET	F	*t*	β	CI (95%) (β)	
									LI	LS
Model 1		0.86	0.73	0.66	4.96	10.18 **	2.11 *			
EI OEA	13.38							0.03	−1.26	1.38
EI UOE	5.35							0.28	−0.28	1.26
EI ROE	7.35							−0.05	−0.92	0.77
EI T	39.79							0.32	−0.43	0.74
IRI TP	1.6							0.21	−0.06	0.76
IRI F	1.36							0.08	−0.24	0.49
IRI PE	1.52							−0.24	−1.06	−0.01
IRI MP	1.49							−0.29	−0.83	−0.09
Model 2		0.80	0.63	0.61	5.30	30.89 **	2.50 *			
EI UOE	1.28							0.63	0.68	1.47
IRI MP	1.28							−0.28	−0.8	−0.07

VIF = variance inflation factor; R^2^ = variance explained by each independent variable; R^2Adj^ = adjusted R-squared; ET = standard error; F = ANOVA statistic; *t =* contrast statistic; β = beta; CI (95%) = 95% confidence interval; LI = lower bound; LS = upper bound; EI OEA = others’ emotions appraisal; EI UOE = use of emotion; EI ROE = regulation of emotion; EI T = total emotional intelligence; IRI TP = perspective taking; IRI F = fantasy; IRI PE = empathic concern; IRI MP = personal distress; * *p* < 0.05; ** *p* < 0.01.

## Data Availability

The data presented in this study are available on request from the corresponding author. The data are not publicly available due to privacy or ethical restrictions.
